# The complete chloroplast genome of a medicinal plant *Anemone flaccida* (Ranunculaceae)

**DOI:** 10.1080/23802359.2020.1763869

**Published:** 2020-05-13

**Authors:** Yuwen Tang, Yifei Liu, Man Liu, Zhigang Hu, Bisheng Huang

**Affiliations:** College of Pharmacy, Hubei University of Chinese Medicine, Wuhan, China

**Keywords:** *Anemone flaccida*, chloroplast genome, phylogeny

## Abstract

*Anemone flaccida* has long-term been used in Chinese traditional medicine with the effects of anticancer, anti-inflammatory, antimicrobial properties, and immune regulation. However, the genomic information of this species is limited, which hinders its further medicinal application. In the present study, the complete chloroplast genome of *A. flaccida* was sequenced and assembled. The genome size was 157,614 bp in length, consisting of a pair of inverted repeat regions (IR, 31,184 bp), a large single copy (LSC, 79,055 bp), and a small single copy (SSC, 16,191 bp). A total of 138 genes were annotated, including 90 protein-coding genes, 40 tRNA genes, and eight rRNA genes. The GC content of the genome was 37.74%. A phylogenetic analysis on the basis of the whole chloroplast genome sequences further suggested a close relationship between *A. flaccida*, *A. narcissiflora*, and *A. trullifolia*. Collectively, the *A. flaccida* chloroplast genome provided new genomic resources which will improve its research and application in the future.

*Anemone flaccida* Fr. Schmidt is a perennial herb belonging to the *Anemone* genus in the Ranunculaceae family. The rhizomes of *A. flaccida*, known as ‘Di Wu’ in Chinese, is considered as a valuable Chinese traditional medicine for treatment of punch injury and rheumatoid arthritis (Liu et al. 2015). Previous studies on *A. flaccida* mainly focused on its chemical components, medicinal activities, and biosynthesis pathway about its main active ingredients (Han et al. 2016; Mo et al. 2019). However, the genomic resource of *Anemone* species was limited and the evolutionary relationship between *A. flaccida* and other *Anemone* species was not well investigated. In the present study, we sequenced and assembled the complete chloroplast (cp)genome of *A. flaccida*, and further investigated the phylogenetic relationship between *A. flaccida* and other representative *Anemone* species. All these will benefit both breeding and medicinal applications of *A. flaccida* in future.

The sample of *A. flaccida* used for sequencing was collected from Changyang County, Hubei province of China (30°17′N, 110°48′E), and the voucher specimen was deposited at the herbarium of Hubei University of Chinese Medicine (specimen code CY170410). The total genomic DNA was extracted from fresh leaves of the sample using a modified CTAB method (Doyle and Doyle 1987). A genomic library with an insert size of 500 bp was constructed and the library was sequenced using the Illumina Hiseq 4000 Sequencing System (Illumina, Hayward, CA) commercially in the Benagen Tech Solutions Company Limited (Wuhan, China). A total of 3.79 Gb of 150 bp paired-end reads were generated. The complete chloroplast genome sequence of *A. flaccida* was assembled using the program SPAdes 3.10.1 (Bankevich et al. 2012). Genome annotation was performed using the DOGMA (Wyman et al. 2004) and tRNAs were identified using the tRNAscan-SE v 1.21 (Schattner et al. 2005).

The complete chloroplast genome sequence of *A. flaccida* (GenBank accession number: MT317179) was 157,614 bp in length, containing a pair of inverted repeat (IR) regions of 31,184 bp each, separated by a large single-copy (LSC) region of 79,055 bp and a small single-copy (SSC) region of 16,191 bp. The overall GC content of the cp genome was 37.74%, and the corresponding values in LSC, SSC, and IR regions were 35.69, 31.88, and 41.87%, respectively. The circular genome contained 138 genes, including 90 protein-coding genes, 40 tRNA genes, and eight rRNA genes. Among these genes, 13 protein-coding genes, five tRNA genes, and five rRNA genes were duplicated in the IR region. With annotations of genes, 13 genes (atpF, ndhA, ndhB, rpl2, rpoC1, rps12, petB, petD, trnK-UUU, trnV-UAC, trnL-UAA, trnI-GAU, and trnA-UGC) were found to contain one intron, while two genes (clpP and ycf13) possessed two introns.

To investigate the evolutionary relationship of *A. flaccida* with other taxa within the tribe Anemoneae from the Ranunculaceae family, the whole chloroplast genome sequences of a panel of 16 plants, including 10 *Anemone* plants, two *Naravelia* plants, two *Clematis* plants and one *Anemoclema* plant and one *Pulsatilla* plant were used to construct a tree. Genome sequences were downloaded from the GenBank database and were aligned using MAFFT version 7 (Katoh and Standley 2013). We used MEGA 6 to generate a neighbour-joining tree (Tamura et al. 2013). As shown in the phylogenetic tree (Figure 1), *Anemone* species from the subgenus *Anemone* and the subgenus *Anemonidium* were grouped into two clusters respectively, in which *A. flaccida* was closely related to both *A. narcissiflora* and *A. trullifolia*.

**Figure 1.**
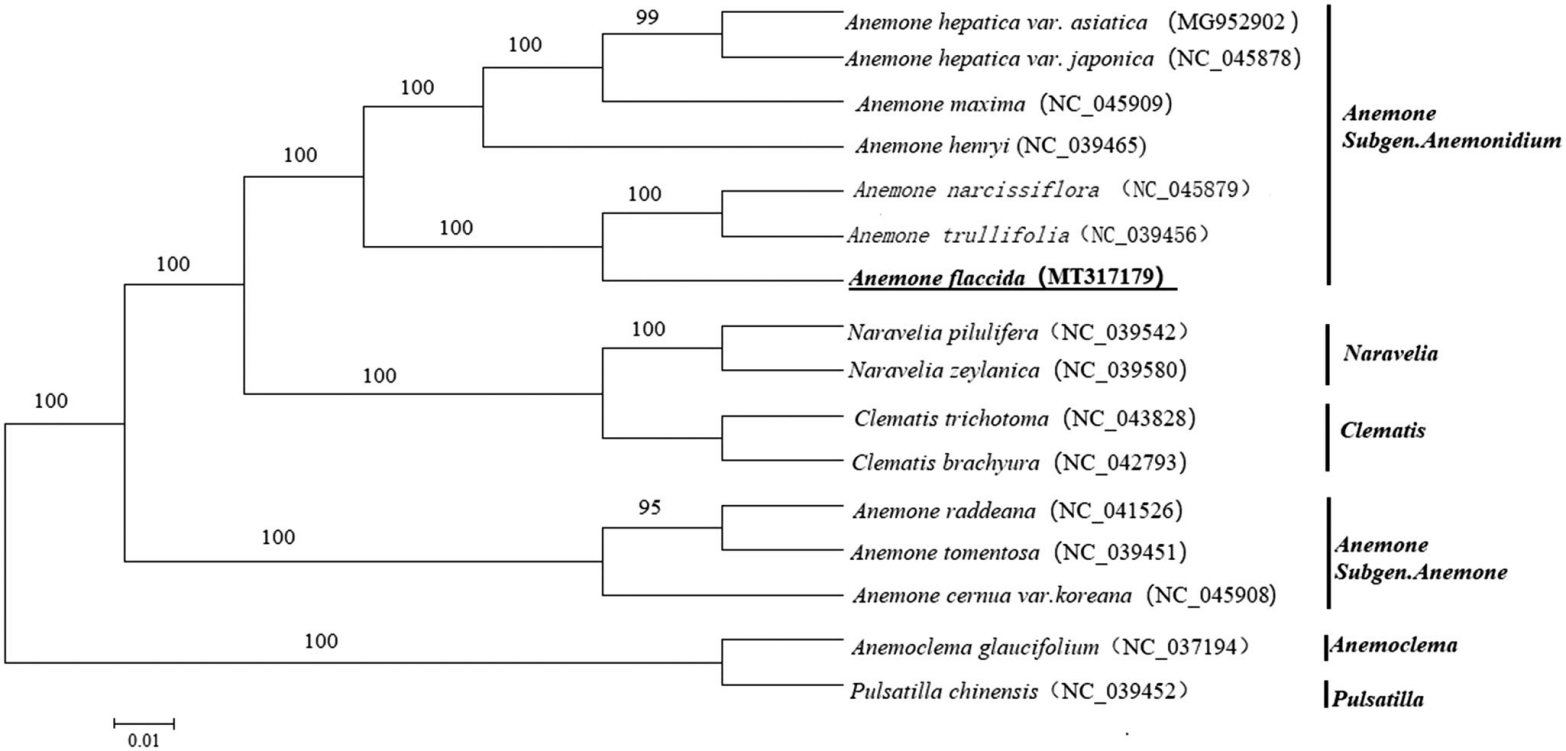
NJ tree based on 16 complete chloroplast genomes of Anemoneae species. The species *A. flaccida* is highlighted in bold.

## Data Availability

The data that support the findings of this study are openly available in NCBI(National Center for Biotechnology Information) at https://www.ncbi.nlm.nih.gov/, reference number MT317179.
